# Efficacy of a Novel Mechanical Cervical Dislocation Device in Comparison to Manual Cervical Dislocation in Layer Chickens

**DOI:** 10.3390/ani9070407

**Published:** 2019-07-01

**Authors:** Rathnayaka M.A.S. Bandara, Stephanie Torrey, Patricia V. Turner, Alex zur Linden, Anna Bolinder, Karen Schwean-Lardner, Tina M. Widowski

**Affiliations:** 1 Department of Animal Biosciences, University of Guelph, Guelph, ON N1G 2W1, Canada; 2 Department of Livestock Production, Faculty of Agricultural Sciences, Sabaragamuwa University of Sri Lanka, Belihuloya 70140, Sri Lanka; 3 Department of Pathobiology, University of Guelph, Guelph, ON N1G 2W1, Canada; 4 Department of Clinical Studies, University of Guelph, Guelph, ON N1G 2W1, Canada; 5 Department of Animal Care Services, University of Guelph, Guelph, ON N1G 2W1, Canada; 6 Department of Animal and Poultry Science, College of Agriculture and Bioresources, University of Saskatchewan, Saskatoon, SK S7N 5A8, Canada

**Keywords:** poultry welfare, brain stem reflexes, brain trauma, cervical dislocation, on-farm euthanasia

## Abstract

**Simple Summary:**

On-farm euthanasia of diseased or injured chickens is a common task within the poultry industry. For animal welfare, the aim of any euthanasia technique is to achieve rapid loss of sensibility, for the process to cause minimal pain, and for death to follow quickly. Manual cervical dislocation (separating the skull from the spine by hand) is a common method for killing poultry on farms, but it can be aesthetically displeasing. Therefore, different tools for neck dislocation (separating the skull from the spine by mechanical device) are developed as alternative euthanasia methods. These tools need scientific assessment for their effectiveness and humaneness. The Koechner Euthanasia Device (KED) (Koechner MFG. CO., INC, USA) is commercially available as a mechanical cervical dislocation tool for poultry. We compared the efficacy of KED with manual cervical dislocation based on time to brain death (irreversible insensibility) and degree of damage to the brain and neck in anesthetized chickens. The anesthetic agents reduced any distress and pain associated with the killing technique. Our results indicated that KED resulted in less damage to the brain, causing longer times to brain death and cardiac arrest in comparison to manual cervical dislocation. We suggest that manual cervical dislocation is more efficient and humane for layer chicken euthanasia than KED.

**Abstract:**

The main objective of this study was to assess the efficacy of mechanical cervical dislocation using the Koechner Euthanasia Device Model C (KED) in comparison to manual cervical dislocation in layer chickens. Laying hens and/or roosters in three different age groups (12, 27–29, and 65–70 weeks old) were randomly assigned to one of three experimental groups: manual cervical dislocation in conscious birds (CD), manual cervical dislocation in anesthetized birds (aCD), or mechanical cervical dislocation by KED in anesthetized birds (aMCD). Anesthetized birds received an intramuscular dose of 0.3 mg/kg medetomidine and 30 mg/kg of ketamine to achieve clinical anesthesia. A comparison of CD vs. aCD responses confirmed that the anesthetic plane abolished or reduced clonic convulsions, nictitating membrane reflex, tonic convulsions, and cloacal relaxation. Time to loss of the pupillary light reflex (~123 s), and time to cardiac arrest (~172 s) were longer (*p* < 0.001) in the birds in the aMCD group than aCD (~71 and ~137 s, respectively). Radiographs revealed that the majority of the birds killed by manual cervical dislocation (CD + aCD) had dislocations between the skull and atlas (C1) or between cervical vertebrae C1–C2. The KED resulted in a majority of dislocations at C2–C3. Birds killed by manual cervical dislocation presented more subdural and parenchymal hemorrhage in the brain stem compared to birds killed by KED. Radiographs indicated the presence of fractures in a few birds killed by either method (CD + aCD versus aMCD). Compared to manual CD, KED resulted in less brain trauma and a longer latency to brain death, indicating a lower efficacy of KED as an on-farm killing method.

## 1. Introduction

Over the last decade, techniques used to kill poultry on farms have come under public and scientific scrutiny due to concern for animal welfare. For the welfare of the animal, the goal of any euthanasia technique is to achieve insensibility as quickly as possible, for the process to cause minimal pain, and for death to follow quickly. Manual cervical dislocation is the most common method for killing poultry on farms. Manual cervical dislocation involves stretching and separating the cervical vertebrae by hand, rupturing the blood vessels, and causing death by cerebral ischemia and extensive damage to the spinal cord and brainstem [1,2,3]. Optimal application of cervical dislocation separates the cervical vertebrae between the skull and the first cervical vertebra (C1) and completely transects the spinal cord [4]. However, cervical dislocation may be esthetically displeasing to personnel performing it. Additionally, the use of manual cervical dislocation to kill poultry on-farm has been restricted to birds weighing less than 3 kg and to 70 birds per person per day through European Union (EU) legislation [5].

To address the limitations around manual cervical dislocation, different tools have been developed to perform cervical dislocation mechanically. Some devices (e.g., killing cone and heavy stick) involve stretching, whereas others attempt to dislocate the cervical vertebrae by forcing a blunted edge between two vertebrae (e.g., pliers, and Burdizzo) without stretching or twisting the neck [6]. Both manual and mechanical cervical dislocation methods have been questioned for humaneness based on evidence that loss of sensibility is not immediate in poultry species [1,6]. In their guidelines, the American Veterinary Medical Association (AVMA) [7] specifies that cervical dislocation “must result in luxation of the cervical vertebrae without primary crushing of the vertebrae and spinal cord”. However, there is evidence that vertebral separation with the aid of a tool can cause fractures to the cervical vertebrae in chickens [3] and turkeys [3,8,9].

At present, different devices are manufactured for cervical dislocation and marketed to provide poultry producers with options for on-farm euthanasia. However, there is a lack of evidence on the different devices’ efficacy in causing a humane death. The Koechner Euthanizing Device (KED; Koechner Mfg. Co., Inc. Tipton, MO, USA) is commercially available for poultry as a mechanical cervical dislocation device. Woolcott et al. [8] assessed the efficacy of the KED (Model S designed for chicks and poults) in anesthetized poults and young turkeys. Their results revealed longer times to cessation of brain stem reflexes and more fractures in cervical vertebrae in the birds killed by KED-S in comparison to manual cervical dislocation. Jacobs et al. [10] also found longer times to loss of brain stem reflexes when broiler chickens were killed by KED-model-C compared to manual CD. Therefore, there is a need to evaluate the KED in layer chickens before recommending its use on farms.

Ethical concerns arise in the evaluation of novel killing devices in conscious animals. Therefore, it is necessary to initially evaluate novel killing devices either using cadavers [11] or anesthetized animals [8]. The use of anesthetic agents reduces the distress and pain associated with killing techniques by abolishing the awareness of the animal. Anesthetics produce insensibility by preventing integration (blocking the interactions among specialized brain regions) or by reducing information (reducing the number of activity patterns available to cortical networks) [12]. Efficacy and humaneness of physical killing methods in poultry are often assessed based on time to loss of brain stem reflexes and degree of physiological trauma that is likely to result in rapid death [8,13,14,15,16]. Previous studies have reported some brain stem reflexes present under anesthesia in poultry, whereas others have not [17]; thus, there is a need to compare conscious and anesthetized manual CD to understand the effects of anesthesia on the measures used to determine efficacy.

The main objective of the current study was to assess the efficacy of mechanical cervical dislocation using the Koechner Euthanasia Device (KED model-C) in comparison to manual cervical dislocation based on behavioral responses, brain stem reflexes, and postmortem analysis of the physiological damage produced in three different age groups of layer chickens. For ethical reasons, the KED was tested on anesthetized birds and compared with anesthetized birds killed by manual cervical dislocation. In order to determine which measures would be valid for use in anesthetized birds, our secondary objective was to determine the effect of the anesthetic agents used in the study on behavioral and reflex responses by comparing anesthetized and conscious birds killed by manual cervical dislocation.

## 2. Materials and Methods 

The procedures and protocol for this research were reviewed and approved by the University of Guelph Animal Care Committee (AUP 3321) which holds a Good Animal Practice certificate 101 issued by the Canadian Council on Animal Care.

### 2.1. Animals and Facilities

The birds were obtained from different research projects that were conducted at the Arkell poultry research station of the University of Guelph. All the birds were targeted for euthanasia as they had reached scheduled end of study or because of routine flock depopulation. Different strains and age groups of birds were available on different days. A health assessment was performed on the birds prior to the experiment. Birds suspected of having disease or physical injuries were excluded. Laying hens and/or roosters in three different age groups (12, 27–29, and 65–70 weeks old) were randomly assigned to either manual cervical dislocation (CD), anesthetized manual cervical dislocation (aCD), or anesthetized mechanical cervical dislocation by KED (aMCD). Order of application of assigned treatments on a trial day was determined by using the random number generator in Excel. Sex was not balanced among the treatments due to the disproportionate availability of female and male layer chickens in the different age groups. A total of 72 chickens were enrolled in this study: Eight birds in each age group were evaluated with each of the three killing methods. Strain, sex, body weight, and sample sizes for the different age classes of birds used in the study are given in Table 1. 

### 2.2. Koechner Euthanizing Device (KED)

**Figure 1:**
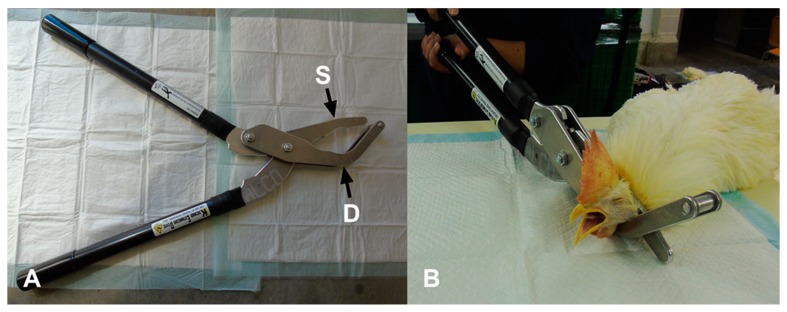
(**A**) Koechner euthanasia device (KED model-C): S—single side blade; D—double angle blade. (**B**) Application of KED model-C in a 65-week-old anesthetized rooster.

The Koechner Euthanizing Device (KED) is manufactured as a mechanical cervical dislocation device for poultry by Koechner MFG. CO., INC (2016) (U.S. Patent No. 8,152,605,). Four different commercial models of the KED are manufactured for different weight classes [18]. The model-S is designed for birds weighing up to 1.8 kg, and the model-T is for birds weighing up to 20.5 kg. The model-Txl is designed for birds weighing over 29.5 kg. The KED model evaluated in the current study was the model-C consisting of a 69 cm handle, marketed for birds weighing up to 13.5 kg (Figure 1A).

### 2.3. Anesthesia and Killing Procedures

Anesthesia was induced in the birds by using a combination of medetomidine (1 mg/mL) (CepetorTM, DIN:02337177, Modern Veterinary Therapeutics, LLC, Miami, FL, USA) and ketamine (100 mg/mL) (Ketaset^®^, DIN: 02173239, Pfizer Animal Health, Kirkland, QC, CA). Appropriate drug doses to induce surgical anesthesia in chickens were established in a pilot study. The chosen doses (medetomidine = 0.3 mg/kg body weight; ketamine = 30 mg/kg body weight) were administered as separate injections into the breast muscle by a veterinarian monitoring the anesthesia.

After injecting the drugs, the birds were kept in a crate in a quiet and dark room for ten minutes for the anesthesia to take effect. After another 5 min without interference, breathing, heartbeat, and pedal reflex were assessed. Fifteen minutes after the drug application, birds were assessed for breathing, pupillary reflex, pedal reflex, neck muscle tone, jaw tone, and heartbeat. The same responses were checked directly prior to application of the killing method. Birds were determined to be ready for the killing method when they were non-responsive to handling and when assessment of breathing pattern, heart auscultation, jaw tone, and pedal reflex by the monitoring veterinarian indicated surgical anesthesia.

Manual cervical dislocation was performed by a trained and experienced technician. The bird’s head was held in the operator’s palm, with the neck between the index finger and thumb. Manual cervical dislocation was performed in one swift movement with the operator pulling down on the bird’s head, stretching the neck, while rotating the bird’s head upwards into the back of the neck.

A trained and experienced technician applied the KED device. The KED was applied according to the manufacturer’s instructions (Koechner MFG. Co., INC. 2016). Prior to the experiment, the KED device was applied to cadavers in different age groups and radiographs were used to confirm correct placement and pressure. All of the birds were manually restrained on a table in a sternal recumbent position when applying the KED. The double angle blade was placed under the neck of the bird while the single side blade was placed dorsally above the top of the neck at the base of the skull. The handles were brought together quickly and firmly to cause dislocation (Figure 1B). Then, the device was removed from the neck of the bird.

### 2.4. Antemortem Assessment

The measures used in the antemortem assessment are described in Table 2. Each measure was checked immediately after application of the killing method and then at 10-s intervals until cessation. The presence of a heartbeat was monitored through a stethoscope, and cardiac arrest was determined to have occurred when no discernible heartbeat could be heard. All of the procedures and responses of each bird were video recorded. Time to first feather erection was based on blinded video recordings. The other measures were collected unblinded by live observations, and the time at each event was confirmed with the video records.

### 2.5. Postmortem Assessment

#### 2.5.1. Assessment of Radiographs

Radiographs were performed on all birds immediately following euthanasia in an adjacent room. In order to prevent further damage to the cervical dislocation site, each bird was carried by hand in the horizontal plane to prevent movement of neck and head. Four views were performed of each bird (dorsal ventral, ventral dorsal, right lateral, and left lateral) using a portable x-ray unit (Poskom VET-20BT X-ray unit with 80 kVp, 2.5 mAs power). The birds were placed directly on the wireless direct digital imaging receptor panel (ULTRAMAXX, Promark imaging; 19.65 x 23.6 cm image matrix, a-SI TFT-PIN (thin filament transistors), 77-micron pixel pitch, 6.0 to 7.0 lp/mm resolution) for radiograph acquisition.

Radiographs were assessed for site of luxation/subluxation and for presence, type, and site(s) of fractures (Table 3) by a board-certified veterinary radiologist blinded to the treatment. Digital Imaging and Communications in Medicine (DICOM) formatted radiographs were interpreted after importing the images into a DICOM viewer (Osirx MD, version 8.0.2, Pixmeo SARL, Bernex, Switzerland).

#### 2.5.2. Macroscopic Assessment of Tissue Damage

Degree of external damage and bleeding caused by each killing method was assessed (0–2 scale) based on laceration of the skin and the presence of external hemorrhage on the neck: 0 = no laceration of the skin, 1 = laceration of the skin with no external bleeding, and 2 = laceration of the skin with external bleeding [15].

Dissection was performed on all birds to assess the degree of subcutaneous hemorrhage (SCH) at the site of cervical dislocation, damage to the trachea, transection of the spinal cord, and degree of subdural hemorrhage (SDH) on the brain. Scoring criteria for macroscopic SCH and SDH are presented in Table 4 [8,15,16]. SCH was scored by excising the skin around the neck of the bird to measure the degree of hemorrhage at the site of cervical dislocation. The skull was lifted, and dura were removed to assess the SDH on the brain.

#### 2.5.3. Microscopic Assessment of Brain Trauma

Once the macroscopic assessment was completed, brains and a sample of spinal cord from the site of cervical dislocation were collected from six randomly selected birds in each age group per killing method for microscopic evaluation. The brains and spinal cord samples were placed in 10% buffered formalin for at least 14 days before trimming. For consistency, all trimming was performed by one individual. Three sections of the brain (cerebrum, mid brain and thalamus, and cerebellum/hind brain) and a section of the spinal cord at the site of cervical dislocation was sampled [8]. The tissue sections were embedded in paraffin, cut at 4 µm, and stained with hematoxylin and eosin using standard techniques to make microscopic slides (Animal Health Laboratory, University of Guelph). The sections on the slides were assessed microscopically by a veterinary pathologist blinded to the treatments to determine the degree of SDH and parenchymal hemorrhage (PCH). The degree of SDH and PCH were scored for each microscopic slide using the same procedure as Woolcott et al. [8,16] and Bandara et al. [15] (Table 4).

### 2.6. Statistical Analyses

Statistical analyses were conducted using SAS 9.4 (SAS Institute Inc., Cary, NC, USA). Two separate statistical analyses were used using the same model described below. The first analysis was used to compare the behavioral responses and reflex variables of anesthetized birds killed by manual cervical dislocation (aCD) to conscious birds killed by manual cervical dislocation (CD) in order to determine the effects of anesthesia on each outcome variable. In the second analysis, the response variables of the aCD were statistically compared to those of the anesthetized birds killed by KED (aMCD).

Fisher’s exact tests were performed on the birds pooled across age groups that showed pupillary light reflex, nictitating membrane reflex, gasping, feather erection, clonic convulsions, tonic convulsions, and cloacal relaxation to find the effect of anesthesia (CD vs. aCD) and killing method (aCD vs. aMCD). The numbers of birds exhibiting luxation, fractures, macroscopic SDH, and transection of spinal cord were similarly analyzed by Fisher’s exact tests.

Generalized linear mixed models (GLMM) were used to analyze the fixed effects of the killing method, age, and killing method by age interaction of selected antemortem measurements (time to loss of pupillary light reflex, duration of gasping, time at first feather erection, and time to cessation of heartbeat). Nictitating membrane reflex, clonic convulsion, tonic convulsion, and cloacal relaxation were excluded from the analysis due to a low number of birds exhibiting these measures in the anesthetized groups. A least significant means separation was conducted by using the Tukey–Kramer test.

Generalized linear mixed models (GLMM) with multinomial distribution and cumulative logit link functions were used to analyze the effect of the killing method, age, and their interaction on postmortem macroscopic SCH at the site of dislocation, microscopic SDH and PCH of the brain and spinal cord, and dislocation site in the neck (multinomial ordinal data). Odds ratios were computed to compare differences in the levels of fixed effects. Data from the three sections of brain from each bird were pooled, and the highest score of the three sections of each brain was used for SDH and PCH analyses [8,15,16]. The same procedure was applied to the SDH and PCH analyses for the spinal cord. Dislocation site was analyzed categorically (skull–C1 = 1; C1–C2 = 2; C2–C3 = 3; etc.); luxation and subluxation were pooled for the analyses.

## 3. Results

### 3.1. Assessment of Antemortem Measures

#### 3.1.1. Effects of Anesthesia on Birds Killed by Manual Cervical Dislocation

The number of birds exhibiting antemortem measures following application of the killing methods are given in Table 5. All conscious and anesthetized birds demonstrated pupillary light reflex before application of the killing method. Pupillary light reflex was observed in all conscious and anesthetized birds after application of the killing method, indicating that there was no anesthesia effect on pupillary light reflex. All conscious birds had a nictitating membrane reflex before application of manual cervical dislocation, but nictitating membrane reflex was present in only one anesthetized bird before application of manual cervical dislocation (*p* = 0.001).

The number of birds observed gasping was not different between conscious birds killed by manual CD (75%) and anesthetized birds killed by manual CD (45%, *p* = 1). The anesthetic did not affect the occurrence of feather erection in conscious birds killed by manual CD (conscious manual CD = 87%, and anesthetized manual CD = 83%, *p* = 1). Some anesthetized birds killed by manual CD did not exhibit clonic convulsions, tonic convulsions, or cloacal relaxation, indicating that there was anesthetic effect on these measures (Table 5). The number of birds exhibiting clonic convulsions was significantly lower (*p* < 0.001) in the anesthetized manual CD group than in the conscious manual CD group. Clonic convulsions with severe wing flapping and paddling were observed in all conscious birds killed by manual CD. However, in 50% of anesthetized birds killed by manual CD, clonic convulsions were absent except at the time of application of the killing method (considered as no clonic convulsions in the analysis; only 2–3 wing flaps were observed at the time of killing). An effect of anesthesia was found in birds that showed tonic convulsions (*p* < 0.001) and cloacal relaxation (*p* < 0.001).

A comparison of the times to cessation of pupillary light reflex and heartbeat, the time at first feather erection, and the duration of gasping in conscious and anesthetized birds killed by manual CD are presented in Table 6. Durations were calculated for only the birds that showed the responses. Overall, the time to cessation of pupillary light reflex was longer (*p* < 0.05) in conscious birds than in anesthetized birds. There was a significant age by treatment interaction (*p* = 0.03) with a longer duration for the cessation of pupillary light reflex in conscious birds killed by manual CD compared to the anesthetized birds killed by manual CD birds in the 65–70-week group. The duration of gasping did not differ between the conscious and anesthetized groups (*p* = 0.35). Anesthesia did not affect time to first feather erection (*p* = 0.22). There was no difference in the time to cessation of heartbeat in conscious versus anesthetized birds killed by manual CD (*p* = 0.14). Time to cessation of heartbeat took longer in younger birds compared to older ones (*p* < 0.001), but there was no anesthesia-by-age interaction (*p* = 0.59).

#### 3.1.2. Effect of Manual CD Versus Mechanical CD in Anesthetized Birds

The number of anesthetized birds killed by manual CD or mechanical CD and their responses are shown in Table 5. All anesthetized birds, regardless of treatment, presented pupillary light reflex. The number of birds that demonstrated gasping was significantly higher (*p* < 0.001) in anesthetized birds killed by mechanical CD than anesthetized birds killed by manual CD. Feather erection occurred in 83% of anesthetized birds killed by manual CD and in 75% of birds killed by mechanical CD (*p* = 0.72). There was no difference in the number of birds that showed clonic convulsions in anesthetized manual CD and anesthetized mechanical CD groups (*p* = 1). Number of birds exhibiting tonic convulsion (*p* = 1) and cloaca relaxations (*p* = 0.74) was not different in anesthetized birds killed by manual CD and mechanical CD.

Table 7 shows mean latencies to loss of antemortem measures or their durations in anesthetized birds killed by manual CD versus mechanical CD in different age groups. Time to cessation of pupillary reflex (*p* < 0.0001), duration of gasping (*p* = 0.01), time to first feather erection (*p* = 0.04), and time to cessation of heart beat (*p* = 0.01) were all longer in the anesthetized birds killed by mechanical CD compared to anesthetized birds killed by manual CD in all age groups. 

### 3.2. Assessment of Postmortem Measures

#### 3.2.1. Assessment of Cervical Vertebrae Radiographs

Results of radiographic scoring for the presence and site of cervical dislocation are tabulated in Table 8. The cervical vertebrae were assessed for any sites of luxation and/or subluxation. All birds killed by manual CD had luxation (complete dislocation) irrespective of whether they were conscious or anesthetized, whereas only 54% of birds killed by mechanical CD had luxation (*p* < 0.001). The site of dislocation was significantly different for treatment (*p* < 0.001) but not for age (*p* = 0.65) or age-by-treatment interaction (*p* = 0.91) in birds pooled across luxation and subluxation. There was a greater chance of causing dislocation further down the vertebrae than the recommended skull to C1 in chickens killed with mechanical CD than manual CD. Overall, 58% of the birds (conscious and anesthetized) killed by manual cervical dislocation had luxation in between the skull and C1. In contrast, the majority of birds (75%) killed with mechanical CD had luxation or subluxation between the C2 and C3 vertebrae.

**Figure 2:**
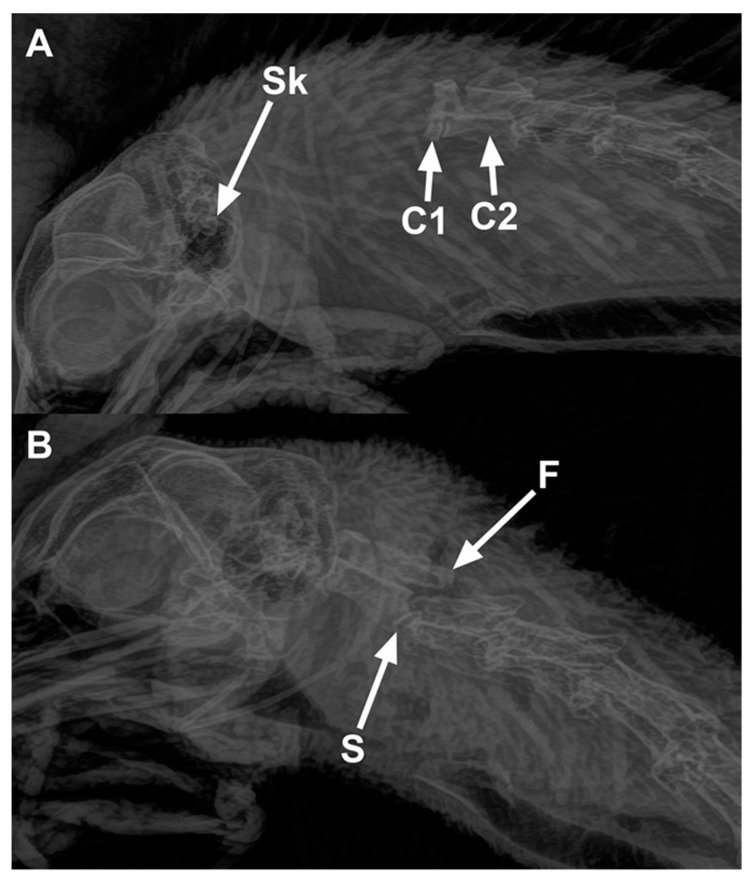
Radiographs of chickens showing cervical dislocations. (**A**) Luxation between the skull and C1 vertebra in a 65-week-old rooster killed by manual cervical dislocation: Sk—Skull, C1—first cervical vertebra, and C2—second cervical vertebra. (**B**) Subluxation between C2 and C3 (letter S shows the site of subluxation) in a 65-week-old rooster killed by KED: fractures (F) are present on the articular processes of the C3 vertebra.

The number of birds scored with fractures and the different types of fractures observed in the cervical vertebrae are presented in Table 9. Fractures were observed in less than 10% of the birds regardless of treatment or whether they presented with luxation or subluxation. There was no difference between manual cervical dislocation and MCD in birds with fractures (*p* = 0.19). Overall, 4 birds out of 24 (17%) killed with MCD and 3 out of 48 (6%) killed by manual cervical dislocation had fractures of the cervical vertebrae. Radiographs demonstrating cervical dislocations (A—cervical luxation, B—cervical subluxation with a fracture) in a chicken are shown in Figure 2.

#### 3.2.2. Macroscopic Tissue Damage Assessment

Spinal cords were transected in all conscious and anesthetized birds killed by manual cervical dislocation in all age groups (Table 8). The number of birds that had transected spinal cords was significantly different between the manual CD group (both conscious and anesthetized) and the mechanical CD group (*p* < 0.001). Spinal cords were intact in 25% of birds killed by mechanical CD and 3/8 in both the 12 and 27–29-week groups. All spinal cords were transected in birds killed by mechanical CD in the 60–75-week group.

Trachea damage was not observed in any of the birds irrespective of the killing method. External skin damage at the site of cervical dislocation was greater in the birds killed with mechanical CD (12 week: 25%; 27–29 week: 75%; 60–65 week: 50%) than the birds killed by manual CD (*p* < 0.001). External skin damage at the site of cervical dislocation was observed in 8% of birds killed by manual CD (conscious and anesthetized combined). Mechanical CD resulted in external bleeding in 33% of the birds pooled across age groups, whereas 4% of the pooled manual CD birds had external bleeding (*p* = 0.002).

Subcutaneous hemorrhage (SCH) at the site of dislocation was different among the treatments (*p* < 0.001), but there was no age effect (*p* = 0.76) or age-by-treatment interaction (*p* = 0.23) (Table 10). Except for one, all conscious and anesthetized birds killed by manual cervical dislocation had a score of 2 or more for SCH. The highest score of 4 was found in over half of the birds killed by manual cervical dislocation (conscious = 54%, anesthetized = 56%). A score of 4 was absent in all birds killed by the mechanical CD device.

Macroscopic subdural hemorrhage (SDH) (score 1) in the brain was observed in only one bird out of 24 (12 weeks old) killed with MCD. Overall, 21% of the birds killed by manual CD (10/48) had macroscopic SDH on the brain (*p* = 0.09).

#### 3.2.3. Microscopic Evaluation

Table 11 gives descriptive statistics for SDH and PCH (all scores > 0) in the brains of birds killed by the different methods in the three age groups. None of the birds in any group had SDH of the cerebrum. Birds killed by manual CD (conscious and anesthetized combined) had SDH both of the mid brain (30%) and the hind brain (63%), whereas only one bird out of 24 killed with mechanical CD had SDH of the hind brain. Parenchymal hemorrhage (PCH) was observed in few birds overall. Six of 36 killed by manual CD had PCH in the hindbrain. No PCH was observed in any of the birds killed by mechanical MCD. Although more birds killed by manual CD showed microscopic damage compared to those killed by MCD, killing method, age, and method × age interaction did not affect brain SDH or PCH scores.

The number of birds with different scores for subdural hemorrhage (SDH) and parenchymal hemorrhage (PCH) in spinal cord are presented in Table 12. Over 80% of all birds sampled had SDH of the spinal cord (method *p* = 0.16), but there was an effect of age (*p* < 0.01) and a tendency for an age-by-killing-method interaction (*p* = 0.06). Birds at 12 weeks had 22 times greater chance of having lower scores than birds at 65–70 weeks and 6 times greater chance of having lower scores than birds at 27–29 weeks. Birds at 27–29 weeks had 4 times greater chance of having lower scores for SDH of the spinal cord than birds at 65–70 weeks. There was a trend of having lower scores of SDH of the spinal cord in the birds killed by mechanical CD in younger age (12–29 weeks) groups. All the birds killed by mechanical CD had a score of 3 or 4 at 65–70 weeks. PCH in the spinal cord was significantly different among the killing methods (*p* < 0.05). Age and method x age interaction tended to be significant for the PCH (*p* = 0.09 and *p* = 0.07 respectively). Conscious and anesthetized birds killed by manual CD had a greater chance of having lower scores for PCH than birds killed by mechanical CD. Of the 18 anesthetized birds killed by mechanical CD, 13 birds (72%) had mild (score 2) to marked (score 4) PCH. Mild to marked PCH in the spinal cord was found in 50% of birds in the conscious manual CD group and in 27% of the anesthetized manual CD group.

## 4. Discussion

Due to ethical concerns about testing the mechanical device on conscious birds, general anesthesia was induced by using a combination of medetomidine and ketamine in order to abolish any pain associated with the killing technique. Brain stem reflexes, behavioral responses, and physiological parameters could be affected by the anesthetics used, which is why we chose to study both the effect of the anesthesia and the effect of the method.

In the current study, medetomidine was used in combination with ketamine. Medetomidine is rapidly absorbed, causing reliable sedation, analgesia, muscle relaxation, and anxiolysis [19]. However, medetomidine decreases cerebral blood flow [20,21] and heart rate [22]. Thus, medetomidine is combined with other anesthetics to reduce the side effects [19]. Ketamine is often used in combination with medetomidine for avian anesthesia [23]. The major effects of ketamine administration involve the central nervous system. Ketamine can affect eye reflexes and degree of muscle tone and poses a problem when assessing the level of sedation or anesthesia [23]. Therefore, our anesthesia protocol had the potential to affect both antemortem measures (eye reflexes and heart rate) and postmortem measures (brain hemorrhage) and needed to be evaluated.

The anesthetic protocol used did not abolish the pupillary light reflex, which allowed us to use this as an indicator of brain stem death in all birds. However, latency to loss of pupillary light reflex was longer when birds were anesthetized. This is different than Woolcott et al. [8] who found that pupillary light reflex was not longer in turkeys using the same anesthesia protocol as in this study. The anesthetics used in the current study also abolished or reduced four of the other observed behavioral responses and reflexes (nictitating membrane reflex, clonic convulsions, tonic convulsions, and cloacal relaxation), precluding their use for comparing killing methods. Woolcott et al. [8] reported that the presence of the nictitating membrane reflex, the presence of clonic convulsions, and the duration of gasping were affected by the same anesthetic protocol in turkeys. Sandercock et al. [17] observed the nictitating membrane reflex until after respiratory arrest and brain death in laying hens anesthetized with sevoflurane and suggested that the longer time to cessation of nictitating membrane reflex may have been due to combined effects of the anesthetic and sedative they used. In the current study, nictitating membrane reflex was not present in many of the chickens as a result of the anesthetic protocol. We previously found that onset of tonic convulsion and cloacal relaxation correlated with cessation of heartbeat in laying hens and can be used as a practical measure of clinical death [15]. However, our current results revealed that both tonic convulsion and cloacal relaxation were reduced in many of the anesthetized birds. Thus, latency to pupillary reflex, nictitating membrane reflex, clonic convulsions, tonic convulsions, and cloacal relaxation were affected by our anesthetic protocol. The anesthetics did not affect time to cessation of heartbeat in chickens. Since latency to loss of pupillary reflex was shortened by the anesthetic, our estimate for time of brain death using the anesthetized bird as a model is conservative.

The main objective of the current study was to assess the efficacy of mechanical cervical dislocation using the KED (mechanical CD) in comparison to manual cervical dislocation (manual CD). The results of the current study reveal that time to loss of pupillary light reflex, an indicator of brain stem death, was longer in the anesthetized birds killed with mechanical CD than by manual CD. Previous studies also reported a shorter latency to loss of eye reflexes in poultry successfully killed with manual cervical dislocation in comparison to birds killed by mechanical CD using the KED (conscious broilers [10]; anesthetized turkeys [8]). Gregory and Wotton [1] reported that birds killed by mechanical CD (using Semark neck pliers) exhibited a longer time to loss of visual evoked responses compared to manual CD and concluded that cervical dislocation by stretching was more effective than cervical dislocation by crushing. Hernandez [24] also reported a longer time to onset of isoelectric electroencephalograph (EEG) indicating brain death in layers (60 weeks of age; anesthesia was induced by isoflurane at a 5% concentration with oxygen via a face mask) killed by mechanical CD using the KED in comparison to manual CD. Results from the current study reveal that time to cessation of heartbeat and time at feather erection were also longer in the anesthetized birds killed by mechanical CD compared to manual CD. Heard [25] suggested that sudden feather erection in anesthetized birds are an indicator of cardiac arrest or reduced blood flow to the heart. In the current study, >75% of birds exhibited feather erection irrespective of the killing method. Although, recent studies reported that there is no statistical association between time at first feather erection and time of cardiac arrest in chickens [15,24], feather erection does occur during the death process. Thus, the delayed feather erection in the birds killed by mechanical CD does correspond to a longer time to death. 

Ideally, cervical dislocation separates the cervical vertebrae in between the skull and the atlas (C1), completely transecting the spinal cord and disrupting blood vessels. In the current study, the vast majority of the birds killed by manual CD had dislocations in between the skull and atlas (C1) or between C1–C2. However, the KED resulted in a majority of dislocations between C2 and C3. Thus, manual CD caused more cranial dislocations than the KED. Previous studies also reported similar results, indicating that manual cervical dislocation resulted in the majority of dislocations between the skull and atlas (C1) in chickens [3,26,27] and turkeys [8]. Martin et al. [27] reported that more cranial dislocation was associated with improved kill success and more rapid loss of reflexes compared to more caudal dislocations for cervical dislocation.

Anatomical damage to the top of the spinal cord and the base of the brain stem is possible with more cranial dislocation, which is associated with spinal cord concussion, neurogenic shock, and loss of consciousness in humans [28,29]. Manual CD resulted in all birds having cervical luxation, more cranial luxation locations, more spinal cord transections, greater SDH and PCH in the brain stem, and shorter time to loss of brain stem death and cardiac arrest. Except for one bird, none of the birds killed with mechanical CD using the KED had SDH or PCH in the brain, indicating that KED did not cause severe brain trauma. In previous studies on turkeys killed with mechanical CD (KED [8]; burdizzo [14]), mild microscopic lesions were also observed in around 25% of birds. It is difficult to explain how these lesions occurred. However, there was still significantly less severe damage compared to the other killing methods. Previous studies suggested that immediate insensibility and irreversible loss of vital functions are associated with SDH and PCH in the brain [3,14]. We suggest the absence of SDH and PCH may be the reason for the observed longer time to loss of pupillary light reflex and clinical death in the birds killed by mechanical CD.

The majority of birds killed by either manual CD or mechanical CD showed transected spinal cords and SDH and PCH in the spinal cord, indicating both methods were sufficient to cause traumatic damage to the spinal cord. The spinal cord was transected in all 65–70-week-old birds killed by mechanical CD irrespective of whether there was luxation or subluxation. This indicates that KED model-C caused severe damage to the cervical area of the spinal cord of 65–70-week-old roosters. Some muscles responsible for inspiration are innervated by cervical nerves and the accessory cranial nerve (CN X1) [30]. Some authors reported evidence that damage to the cervical area of the spinal cord impaired the functioning of these respiratory muscles, resulting in hypoxia due to failures in respiration process and finally causing death in humans [31]. A higher number of birds killed by mechanical CD exhibited a longer duration of gasping in comparison to manual CD. Therefore, we suggest that hypoxia resulting from spinal cord trauma and leading to cerebral ischemia could be a main reason for the observed gasping reflex and death in mechanically cervical dislocated birds; they also had minimal levels of brain trauma.

There were few fractures in cervical vertebrae of the chickens irrespective of the killing method. This is in contrast to previous studies that reported a prevalence of fractures in poultry killed by manual CD and mechanical CD. Woolcott et al. [8] found considerably more fractures in 3-week-old turkeys killed by mechanical CD (KED model-S), with 78% having transverse or comminuted fractures or fragmentation. In their study, turkeys killed via manual CD also had fractures, although fewer (44%) than with KED. Bader et al. [3] also reported different types of fractures (transverse, comminuted, and longitudinal) in chickens by manual cervical dislocation and turkeys killed by mechanical cervical dislocation using a nonpenetrating forcep with three blunt shear arms. One of limitations of the KED device is a mismatch between device size and size of the bird. This indicates that outcomes from MCD devices cannot be generalized across different models of device or across sizes and species of birds. It also suggests that outcomes from manual CD may depend on the operator.

Less external blood loss and fewer skin tears are preferred in the commercial environments due to biosecurity and esthetic concerns. Similar to the findings of Woolcott et al. [8] in turkeys and Jacobs et al. [10] in broiler chickens, the KED resulted in more external skin damage in layer chickens than manual CD. Other important factors when assessing killing methods for practical on-farm use are safety for stock people and ease of training. Cervical dislocation is easy to perform manually, practical in any emergency killing, and can be applied immediately during barn walkthroughs. However, manual CD is associated with a variability in operators’ performance [27]. We assume a person can be easily trained in operating the KED device, and there is little risk associated with a misuse of the device for the operator or technical difficulties.

## 5. Conclusions

Compared to manual cervical dislocation, mechanical CD using the KED caused more external skin trauma but less trauma to the brain with more incomplete dislocations located in the lower regions of the cervical vertebrae. Birds killed with the KED also showed longer times to loss of pupillary light reflex and cessation of heartbeat. Overall, mechanical cervical dislocation by KED resulted in a lower efficacy in comparison to manual cervical dislocation as an on-farm killing method for layer chickens. The anesthetic protocol abolished or reduced clonic convulsions, nictitating membrane reflex, tonic convulsions, and cloacal relaxation in the current study, suggesting that these behavioral responses or reflexes are not useful as an approximate measure of brain death in cases where ketamine and medetomidine have been administered in chickens.

## Figures and Tables

**Table 1:** Strain, sex, body weight, and sample sizes for the different age classes of birds used in the study.

Age (Week)	Killing Method	Number of Birds	Strain	Sex	Body Weight (Kg) (Avg wt ± SD)
12	CD	8	White Leghorn	All females	1.00 ± 0.10
	aCD	8	White Leghorn	All females	0.94 ± 0.09
	aMCD	8	White Leghorn	2 Males + 6 Females	1.02 ± 0.62
27–29	CD	8	White Leghorn	1 Male + 1 Females	1.77 ± 0.03
			Columbian Rock	1 Male + 5 Females	1.75 ± 0.36
	aCD	8	White Leghorn	3 Males + 3 Females	1.79 ± 0.29
			Columbian Rock	2 females	2.03 ± 0.29
	aMCD	8	White Leghorn	3 Males + 4 Females	1.86 ± 0.35
			Columbian Rock	1 female	1.98
65–70	CD	8	White Leghorn	All males	2.16 ± 0.18
	aCD	8	White Leghorn	All males	2.10 ± 0.19
	aMCD	8	White Leghorn	All males	2.29 ± 0.29

CD = Conscious manual cervical dislocation. aCD = Anesthetized manual cervical dislocation. aMCD = Anesthetized mechanical cervical dislocation by KED.

**Table 2:** List of antemortem assessment measures, description, and procedure use recorded in order of observation after application of each killing method [15].

Measures	Description	Procedure
Pupillary light reflex	Constriction of the pupil in response to light	Light from a medical penlight was directed into the eye and pupil constriction was examined
Nictitating membrane reflex	Transient closure of the nictitating membrane in response to mechanical stimulation	The medial canthus of the eye or the cornea was lightly touched with a fingertip
Gasping	Paroxysmal opening of the beak	Visual observation of paroxysmal opening of the beak
Feather erection	Sudden erection of feathers, not in response to external stimuli	Visual observation of first occurrence of feather erection
Clonic convulsions	Rapid, uncoordinated movement of the body and wings	Visual observation of rapid wing flapping and foot paddling
Tonic convulsions	Muscle rigidity with the legs and wings outstretched	Visual observation of the time of onset of legs and neck outstretched
Cloacal relaxation	Cloacal opening following contractions of cloaca	Visual observation for cloacal opening following contractions
Cardiac arrest	Cessation of heartbeat	Auscultation by using a stethoscope

**Table 3:** Definitions for the terminology used in radiograph evaluation.

Terminology	Definition
Luxation	Complete dislocation of two vertebrae at the articular process joints.
Subluxation	Partial (incomplete) dislocation of two vertebrae at the articular process joints.
Sagittal	Fracture parallel to the long axis of the vertebra on midline.
Trans (transverse)	Fracture perpendicular to the long axis of the vertebra.
Articular processes	Fractures of the articular processes of a vertebra.
Dens	Fracture of the dens (tooth-like process that projects from the cranial aspect of the centrum of the axis (C2) to articulate with the atlas (C1).
Crushed vertebral bodies	Multiple fractures of a vertebra that cannot be classified as a fragment, articular process, or dens fracture.

**Table 4:** Gross and microscopic pathology scoring criteria for macroscopic, and microscopic hemorrhage [8,15,16].

Score	Macroscopic	Microscopic
	Subcutaneous or Subdural Hemorrhage	Subdural or Parenchymal Hemorrhage
0	None	None
1	<25% of surface area	Minimal (<5% of section)
2	26–50% of surface area	Mild (5–10% of section)
3	51–75% of surface area	Moderate (11–30% of section)
4	76–100% of surface area	Marked (>30% of section)

**Table 5:** Number of birds exhibiting antemortem measures following application of the killing methods.

Antemortem Measure		Method	
	CD	aCD	aMCD
	*n* = 24	*n* = 24	*n* = 24
Pupillary light reflex	24	24	24
Nictitating membrane reflex	16 ^a^	1 ^b,d^	9 ^c^
Gasping	18	11 ^d^	24 ^c^
Feather erection	21	20	18
Clonic convulsions	24 ^a^	12 ^b^	11
Tonic convulsions	15 ^a^	1 ^b^	1
Cloacal relaxation	24 ^a^	7 ^b^	5

^a,b^ Different superscripts indicate the number of birds exhibiting the measure is different between CD and aCD according to Fishers’ exact tests (*p* < 0.005), ^c,d^ Different superscripts indicate numbers of birds exhibiting the measure is different between aCD and aMCD according to Fishers’ exact tests (*p* < 0.005), CD = Conscious manual cervical dislocation, aCD = Anesthetized manual cervical dislocation, aMCD = Anesthetized mechanical cervical dislocation by KED.

**Table 6:** Mean latencies to or durations of (± SE s) antemortem measures in conscious and anesthetized chickens killed by manual cervical dislocation in different age groups. *p* values are given for effects of age, anesthesia, and age-by-anesthesia interaction.

Antemortem Measure				*p* Value		
	Age (week)	CD	aCD	Age	Anesthesia	Age × Anesthesia
Time to loss of pupillary reflex	12	81 ± 7	70 ± 7	0.85	<0.05	<0.05
	27–29	77 ± 5	80 ± 5			
	65–70	96 ± 7 ^a^	62 ± 7 ^b^			
	Overall	85 ± 3	71 ± 3			
Gasping duration	12	22 ± 7	50	0.15	0.35	0.77
	27–29	57 ± 8	68 ± 8			
	65–70	73 ± 13	74 ± 17			
	Overall	51 ± 7	64 ± 11			
Time to first feather erection	12	82 ± 15	104 ± 16	0.89	0.22	0.37
	27–29	78 ± 19	114 ± 17			
	65–70	93 ± 15	84 ± 11			
	Overall	85 ± 10	101 ± 10			
Time to cessation of heart beat	12	191 ± 20	173 ± 20	<0.001	0.14	0.59
	27–29	122 ± 9	119 ± 9			
	65–70	152 ± 12	119 ± 12			
	Overall	155 ± 3	137 ± 8			

Durations calculated for only the birds that showed the responses. Superscript letters show the significant difference (*p* < 0.05). CD = Conscious manual cervical dislocation. aCD = Anesthetized manual cervical dislocation.

**Table 7:** Mean latencies to or durations of (± SE s) antemortem measures in anesthetized chickens killed by manual or mechanical cervical dislocation in different age groups. *p* values are given for effects of age, method, and age-by-method interaction.

Antemortem Measure				*p* value		
	Age	aCD	aMCD	Age	Method	Age × Method
Time to loss of pupillary reflex	12	70 ± 14	142 ± 14	0.20	<0.001	0.31
	27–29	80 ± 8	118 ± 8			
	65–70	62 ± 10	107 ± 10			
	Overall	71 ± 7 ^b^	123 ± 7 ^a^			
Gasping duration	12	50	117 ± 18	0.53	<0.05	0.73
	27–29	63 ± 13	102 ± 10			
	65–70	70 ± 20	132 ± 16			
	Overall	64 ± 16 ^b^	117 ± 9 ^a^			
Time to first feather erection	12	104 ± 25	146 ± 30	0.70	0.04	0.56
	27–29	114 ± 10	125 ± 11			
	65–70	87 ± 15	135 ± 14			
	Overall	101 ± 12 ^b^	135 ± 11 ^a^			
Time to cessation of heart beat	12	173 ± 23	172 ± 23	0.09	0.01	0.09
	27–29	119 ± 10	153 ± 10			
	65–70	119 ± 13	193 ± 13			
	Overall	137 ± 9 ^b^	172 ± 9 ^a^			

aCD = Anesthetized manual cervical dislocation. aMCD = Anesthetized mechanical cervical dislocation by KED. Durations calculated for only the birds that showed the responses. Superscript letters show the significant difference (*p* < 0.05).

**Table 8:** Presence and location of luxation/subluxation (from radiographs) and spinal cord transections (from macroscopic evaluation) in conscious and anesthetized chickens killed by manual cervical dislocation and KED ^1^. Values indicate number of birds.

			Luxation					Subluxation					Spinal Cord Transection
Age	Method	Total Number of Birds Assessed	Number of Birds with Luxation	Sk–C1	C1–C2	C2–C3	C3–C4	Number of Birds with Subluxation	Sk–C1	C1–C2	C2–C3	C3–C4	Number of Birds
12	CD	8	8	8	0	0	0	0	0	0	0	0	8
	aCD	8	8	7	0	0	1	0	0	0	0	0	8
	aMCD	8	5	2	1	2	0	3 *	1	1	3	0	5
27–29	CD	8	8	1	7	0	0	0	0	0	0	0	8
	aCD	8	8	3	5	0	0	0	0	0	0	0	8
	aMCD	8	5	0	0	5	0	3	0	0	1	2	5
65–70	CD	8	8	4	3	1	0	0	0	0	0	0	8
	aCD	8	8	5	3	0	0	0	0	0	0	0	8
	aMCD	8	3	0	0	3	0	5	0	0	4	1	8

^1^ Number of birds exhibiting luxation and subluxation were pooled for statistical analysis. Results of statistical analyses are given in the text. CD = Conscious manual cervical dislocation. aCD = Anesthetized manual cervical dislocation. aMCD = Anesthetized mechanical cervical dislocation by KED. * Two birds had subluxations in multiple places.

**Table 9:** Results of radiographic scoring on number of birds with fractures and the types and locations of fractures in conscious and anesthetized chickens.

Age (Weeks)	Treatment	Number of Birds with Fractures	Fracture Type	Location
12	CD (*n* = 8)	0	-	
	aCD (*n* = 8)	0	-	
	aMCD (*n* = 8)	1	Trans	C3
		1	Crushed vertebral bodies	C3 and C4
27–29	CD (*n* = 8)	1	Small fractures	Dens of C2
	aCD (*n* = 8)	1	Trans	Dens of C2
		1	Small fractures	Dens of C2
	aMCD (*n* = 8)	0	-	
65–70	CD (*n* = 8)	0	-	
	aCD (*n* = 8)	0	-	
	aMCD (*n* = 8)	2	Articular processes	C3

CD = Conscious manual cervical dislocation. aCD = Anesthetized manual cervical dislocation. aMCD = Anesthetized mechanical cervical dislocation by KED.

**Table 10:** Macroscopic evaluation of subcutaneous hemorrhage (SCH) at the site of dislocation. Number of birds with each score are indicated.

	Method	Age			Score				*p*	Value
			0	1	2	3	4	Method	Age	Method *Age
SCH	CD	12	0	0	1	2	5	<0.001	0.76	0.23
		27–29	0	0	1	3	4			
		65–70	0	1	3	0	4			
	aCD	12	0	0	2	2	4			
		27–29	0	0	0	0	8			
		65–70	0	0	2	5	1			
	aMCD	12	0	5	2	1	0			
		27–29	0	2	0	6	0			
		65–70	0	2	5	1	0			

CD = Conscious manual cervical dislocation. aCD = Anesthetized manual cervical dislocation. aMCD = Anesthetized mechanical cervical dislocation by KED.

**Table 11:** Summary of microscopic scoring of brains for trauma following application of each of the three killing methods in layer chickens. Number of birds with hemorrhage (any score > 0) in each section are indicated.

Age	Method	Subdural Hemorrhage			Parenchymal Hemorrhage		
(Week)		Cerebrum	Mid Brain	Hind Brain	Cerebrum	Mid Brain	Hind Brain
**12**	CD	0	0	5	0	0	1
	aCD	0	0	4	0	0	0
	aMCD	0	0	0	0	0	0
**27–29**	CD	0	5	6	0	0	2
	aCD	0	1	3	0	0	2
	aMCD	0	0	1	0	0	0
**65–70**	CD	0	1	4	0	0	0
	aCD	0	4	0	0	0	1
	aMCD	0	0	0	0	0	0

Randomly selected 6 brains were assessed for each device in each age group. CD = Conscious manual cervical dislocation. aCD = Anesthetized manual cervical dislocation. aMCD = Anesthetized mechanical cervical dislocation by KED.

**Table 12:** Overall summary of microscopic scoring of subdural hemorrhage and parenchymal hemorrhage in the spinal cord of layer chickens killed by three killing methods. Number of birds with each score are indicated.

	Method	Age			Score				*p*	Value
			0	1	2	3	4	Method	Age	Method *Age
SDH of	CD	12	4	2	0	0	0	0.17	<0.001	0.06
Spinal		27–29	1	1	2	2	0			
cord		65–70	0	0	2	2	2			
	aCD	12	2	0	2	1	1			
		27–29	0	0	2	3	1			
		65–70	0	2	1	2	1			
	aMCD	12	1	3	1	1	0			
		27–29	2	0	4	0	0			
		65–70	0	0	0	4	2			
PCH in	CD	12	3	1	1	0	1	<0.05	0.09	0.07
spinal		27–29	2	1	2	2	0			
cord		65–70	2	0	2	1	0			
	aCD	12	2	0	1	3	0			
		27–29	3	3	0	0	0			
		65–70	2	3	0	1	0			
	aMCD	12	0	2	0	3	0			
		27–29	3	0	4	0	0			
		65–70	0	0	1	3	2			

Randomly selected 6 brains with spinal cords were assessed for each killing method in each age group. CD = Conscious manual cervical dislocation. aCD = Anesthetized manual cervical dislocation. aMCD = Anesthetized mechanical cervical dislocation.
